# PR1P, a VEGF-stabilizing peptide, reduces injury and inflammation in acute lung injury and ulcerative colitis animal models

**DOI:** 10.3389/fimmu.2023.1168676

**Published:** 2023-04-28

**Authors:** Avner Adini, Victoria H. Ko, Mark Puder, Sharon M. Louie, Carla F. Kim, Joseph Baron, Benjamin D. Matthews

**Affiliations:** ^1^ Vascular Biology Program, Children’s Hospital Boston and Harvard Medical School, Boston, MA, United States; ^2^ Department of Medicine, Boston Children’s Hospital, Boston, MA, United States; ^3^ Department of Surgery, Boston Children’s Hospital, Boston, MA, United States; ^4^ Stem Cell Program and Divisions of Hematology/Oncology, Boston Children’s Hospital, Boston, MA, United States; ^5^ Janus Biotherapeutics, Inc, Wellesley, MA, United States

**Keywords:** PR1P, ARDS, Acute Lung Injury, (ALI), ulcerative colitis, inflammation, VEGF

## Abstract

Acute Respiratory Distress Syndrome (ARDS) and Ulcerative Colitis (UC) are each characterized by tissue damage and uncontrolled inflammation. Neutrophils and other inflammatory cells play a primary role in disease progression by acutely responding to direct and indirect insults to tissue injury and by promoting inflammation through secretion of inflammatory cytokines and proteases. Vascular Endothelial Growth Factor (VEGF) is a ubiquitous signaling molecule that plays a key role in maintaining and promoting cell and tissue health, and is dysregulated in both ARDS and UC. Recent evidence suggests a role for VEGF in mediating inflammation, however, the molecular mechanism by which this occurs is not well understood. We recently showed that PR1P, a 12-amino acid peptide that binds to and upregulates VEGF, stabilizes VEGF from degradation by inflammatory proteases such as elastase and plasmin thereby limiting the production of VEGF degradation products (fragmented VEGF (fVEGF)). Here we show that fVEGF is a neutrophil chemoattractant *in vitro* and that PR1P can be used to reduce neutrophil migration *in vitro* by preventing the production of fVEGF during VEGF proteolysis. In addition, inhaled PR1P reduced neutrophil migration into airways following injury in three separate murine acute lung injury models including from lipopolysaccharide (LPS), bleomycin and acid. Reduced presence of neutrophils in the airways was associated with decreased pro-inflammatory cytokines (including TNF-α, IL-1β, IL-6) and Myeloperoxidase (MPO) in broncho-alveolar lavage fluid (BALF). Finally, PR1P prevented weight loss and tissue injury and reduced plasma levels of key inflammatory cytokines IL-1β and IL-6 in a rat TNBS-induced colitis model. Taken together, our data demonstrate that VEGF and fVEGF may each play separate and pivotal roles in mediating inflammation in ARDS and UC, and that PR1P, by preventing proteolytic degradation of VEGF and the production of fVEGF may represent a novel therapeutic approach to preserve VEGF signaling and inhibit inflammation in acute and chronic inflammatory diseases.

## Introduction

ARDS and UC are two common diseases characterized by tissue injury, VEGF dysregulation and severe inflammation. ARDS is an acute and fulminant form of respiratory failure characterized by lung inflammation, hypoxemia and multi-organ failure requiring mechanical ventilation and prolonged ICU hospitalization (1, 2). There are no medical therapies to prevent or cure ARDS and management of ARDS remains supportive (3). UC is similarly characterized by chronic inflammation within the gastro-intestinal tract, and is associated with weight loss, abdominal pain, recurrent diarrhea, and bleeding (4). Treatments for UC include immunomodulatory medicines that are often insufficient to control disease. Proctocolectomy surgery remains the only curative option and is utilized in 15% of all UC patients (5). Neutrophils are the most abundant white blood cells in humans and are the first circulating inflammatory cell to arrive at sites of tissue injury during acute inflammation such as seen in ARDS and UC (6). Upon neutrophil activation, neutrophil-derived proteases including elastase, plasmin, MMP-8, MMP-9, and pro-inflammatory cytokines, including tumor necrosis factor α (TNF-α), interleukin (IL)-1β, and IL-6, are released at the sites of inflammation leading to structural local tissue damage and amplification of the inflammatory response. Further recruitment of neutrophils and other inflammatory cells including lymphocytes and macrophages during ARDS (7, 8) and UC (9, 10) ensues, which in turn contributes to heightened inflammation, the production of reactive oxygen species (ROS) (11) with disruption of cell membranes and ongoing tissue damage. Curiously, neutrophils and other inflammatory cells are also required to prevent invasion of disrupted epithelial cell barriers by infectious pathogens, and so anti-inflammation strategies envisioned to cure or treat ARDS and/or UC must balance their effects on inflammatory cell function and numbers. Also, as noted above, VEGF dysregulation is often associated with tissue injury and inflammation, and a clear understanding of how VEGF might simultaneously mediate inflammation and restoration of tissue to health is not clear.

VEGFs are a family of endogenous growth factors with angiogenic, survival and anti-apoptotic properties (12, 13). VEGF-A is a highly conserved member of the VEGF family, and VEGF_165_ (a 165-amino acid isoform of VEGF-A, henceforth referred to as VEGF) is an ubiquitous angiogenic and survival factor that binds VEGF receptor 1 (VEGFR1) and 2 (VEGFR2), as well as neuropilin-1 (NRP-1) (14). These receptors are on multiple cell types and tissues throughout the body including lung endothelial and epithelial cells, neutrophils and other inflammatory cells that are resident in, or which infiltrate the lungs and GI tract following injury (10, 15). VEGF signaling leads to a wide variety of biological and functional outcomes that are critical in maintenance of cell and tissue health and healing following tissue insults (16–19). Neutrophil derived proteases such as elastase and plasmin cleave VEGF (20–22) into a smaller VEGF isomer (fragmented VEGF (fVEGF)) with altered VEGF signaling (20), and so an important but underrecognized effect of increased neutrophil activity during acute and chronic inflammation such as seen in ARDS (23, 24) and UC (25) is VEGF dysregulation.

We recently developed a 12 amino-acid VEGF binding peptide (PR1P (26)) that protects VEGF from degradation by the proteases elastase and plasmin, and upregulates VEGF signaling (27). We and others have identified potential therapeutic efficacy for PR1P in multiple animal models all characterized by tissue injury with subsequent inflammation and VEGF dysregulation, including in emphysema (27), myocardial infarction (28), nerve degeneration (29), hind-limb and cerebral ischemia (26, 30), ligament regeneration (31), and chronic wounds (32). fVEGF, the by-product of VEGF degradation, was recently shown to be a chemoattractant to macrophages (20, 21). We therefore hypothesized that fVEGF is a chemoattractant to neutrophils, and that by preventing fVEGF production *via* VEGF stabilization, PR1P reduces neutrophil migration into sites of ongoing inflammation all the while upregulating VEGF signaling by preserving local VEGF levels and concentration gradients.

To test this hypothesis, we first designed an *in vitro* neutrophil migration assay to characterize the ability of PR1P to mediate fVEGF induced neutrophil chemoattraction, and then tested the potential of PR1P to mitigate inflammation in lung injury and ulcerative colitis animal models. Here we present data showing that fVEGF is chemoattractant to neutrophils *in vitro*, and that by blocking the production of fVEGF during VEGF proteolysis by elastase, PR1P can mediate VEGF dependent neutrophil chemoattraction. Subsequently, inhaled PR1P reduced neutrophil migration into the airways following injury in three separate murine acute lung injury models including from lipopolysaccharide (LPS), bleomycin and acid. Reduced presence of neutrophils in the airways was associated with decreased pro-inflammatory cytokines (including TNF-α, IL-1β, IL-6, and Myeloperoxiase (MPO)) in broncho-alveolar lavage fluid (BALF). Finally, PR1P prevented weight loss and tissue injury and reduced plasma levels of key inflammatory cytokines IL-1β and IL-6 in a TNBS-induced colitis model in the rat. Together these data demonstrate that fVEGF may play a key role in mediating inflammation in acute lung injury (ALI) and UC, and that PR1P may represent a novel therapeutic approach to stabilize VEGF and preserve VEGF signaling and inhibit inflammation to improve outcome in acute and chronic inflammatory diseases.

## Materials and methods

### Mice

6-week-old female C57BL/6J mice were purchased from Jackson Laboratory (Bar Harbor, ME) and were kept in a pathogen-controlled environment in standard cages and were fed ad libitum. Protocols for the *in vivo* studies were approved by the Institutional Animal Care and Use Committee (IACUC) at Boston Children’s Hospital.

### Rats

Experiments with rats were performed by the CRO Washington Biotechnology Inc. (Simpsonville, MD). Male Sprague-Dawley rats were purchased from Envigo (Dublin, VA). The rats were cared for per company protocol, were examined daily and found to be free of any clinical signs of disease or distress.

### Peptides

The 12-mer peptides PR1P (DRVQRQTTTVVA), scrambled PR1P (VRQVVTARDTTQ) and fVEGF(ARQENPCGPCSERRKHLVQDPQTCKCSCKNTDSRCKARQLELNERTCRCDKPRR) were all synthetized by Genscript (Piscataway, NJ).

### Induction of ulcerative colitis in rats by tri-nitrobenzene sulfonic acid

The tri-nitrobenzene sulfonic acid (TNBS) model is the most commonly used model to investigate the pathogenesis of, and for developing new anti-inflammatory strategies for ulcerative colitis (UC) (33). Colonic damage is created by the intrarectal application of a barrier disrupting ethanol and hapten mixture and since ethanol splits the epithelial layer, it allows the lamina propria to be exposed to bacterial components (34, 35). The rat model was performed by the (CRO) Washington Biotechnology (Simpsonville, MD). Sprague-Dawley male rats (210-230 grams) were randomly divided into five experimental groups with eight rats in each group including: (1) vehicle control, (2) TNBS-treated, (3) TNBS+PR1P (0.5 mg/kg), (4) TNBS+PR1P (5mg/kg), and (5) TNBS+ prednisolone, (50 mg/kg). Rats were deprived of food for 24 hours, anesthetized with intraperitoneal ketamine (80 mg/kg) + xylazine (10 mg/kg) and a polyurethane nutritional cannula was inserted through the anus. To induce acute colitis, 48 mg/kg TNBS dissolved in 0.25 mL 50% ethanol was instilled into the colon through the cannula. Control animals were instilled with vehicle (50% ethanol in saline). To evaluate the potential effects of PR1P in TNBS-induced colitis, rats were treated daily for 7 days with either PR1P, prednisolone or saline as noted above. Body weight, stool consistency and quality (diarrhea, blood) were recorded daily. Rats were sacrificed on day 7, weighed, and blood collected and stored using pre-chilled EDTA-microtainer tubes (Becton-Dickinson),. Blood samples were processed for plasma and stored at -80°C. After a midline incision in the abdomen, the colon was scored for adhesions and stricture. The colon was then removed from the rectum to the ileo-caecal juncture and the colon length recorded. After a longitudinal incision of the colon, the fecal material was removed and the colon weight, colon wall thickness, ulcer length, and ulcer width were recorded. A Colonic Score (maximum = 12) was calculated as follows: Adhesion: (i) none = 0, ii) minimal = 1, iii) involving several bowel loops = 2, Stricture: i) none = 0, ii) minimal = 1, iii) mild = 2, iv) severe, proximal dilatation = 3, Ulcer: i) none = 0, ii) focal hyperemia, no ulcers = 1, iii) ulcer without significant inflammation (hyperemia and bowel wall thickening = 2, iv) ulceration of 1 - ¾ 3 cm = 3, v) ulceration > 3 - < 6 cm = 4, vi) ulceration ≥ 6 cm = 5, Wall thickness: i) less than 1 mm = 0, ii) 1 – 3 mm = 1, iii) > 3 mm = 2. A section of the colon was preserved in 20 volume 10% neutral buffered formalin (Richard-Allan Scientific). The carcasses were disposed of appropriately. The formalin-preserved colon samples were submitted for histopathological processing and evaluation. Each tissue was embedded longitudinally in its own block. One slide per block was sectioned at - 4µm and stained with hematoxylin and eosin (H&E). Slides were evaluated with light microscopy by an ACVP board-certified veterinary pathologist. Histologic findings were diagnosed and were given a severity score of 0- 5 (0 = not present/normal, 1 = minimal, 2 = mild, 3 = moderate, 4 = marked, 5 = severe). Features were scored according to extent as follows: Grade 0: Not present/within normal limits, Grade 1: Minimal; <10% of sample affected, Grade 2: Mild; 10-25% of sample affected, Grade 3: Moderate; 26-50% of sample affected, Grade 4: Marked; 51-75% of tissue affected, Grade 5: Severe; >75% of tissue affected.

### Murine LPS model of acute lung injury

Mice (22 + 2 g) were placed in a whole-animal nebulization chamber (14x5x8cm) and allowed to spontaneously breathe nebulized lipopolysaccharide LPS (salmonella enterica) (Sigma-Aldrich, St. Louis, MO) (0.6mg/mice), followed by PR1P or scrambled peptide (SP) (300 μg/ml in 3 ml of 0.9% normal saline) for 20 minutes (Proneb Ultra II Nebulizer, Pari Respiratory Equipment). Animals were sacrificed 24h after inhalation experiments and lungs were harvested. The lung tissue was minced using surgical scissors and suspended in 3 ml of serum-free bronchial epithelial-cell growth basal medium containing Liberase (2 mg/ml; Sigma-Aldrich), Dispase (5 U/ml; Stemcell Technology), and DNase (50 U/ml; Sigma-Aldrich) for 30 minutes at 37 C. Digested tissue was filtered (70-mm mesh Thermo Fisher Scientific), and filtered cells were washed twice with FACS media (PBS containing 3% FCS) and centrifuged at 1,000 rotations/min for 5 minutes. Pelleted material was incubated for 90 seconds in erythrocyte–lysis buffer (Sigma-Aldrich), resuspended in FACS media, and prepared for FACS analysis.

### Murine bleomycin model of ALI

Mice were anesthetized with ketamine/xylazine (ketamine (125 mg/kg; Sanofi Winthrop Pharmaceuticals, New York, NY), xylazine (12.5 mg/kg; Phoenix Scientific, St. Joseph, MO) and injected intratracheally using a 24-gauge angiocatheter with 100μl sterile PBS or bleomycin (Cayman Chemicals, Arbor, MI, 0.75 mg/kg dissolved in PBS). Four days later, animals were sacrificed and bronchoalveolar lavage fluid (BALF) was collected by instilling 1 mL chilled PBS into the lungs *via* a 24-gauge angiocatheter (Becton Dickinson) and collected. The procedure was repeated 5X each with fresh PBS. The BALF was then centrifuged for 5 min at 300 g. as analyzed for total cell count and leukocyte differential. Cells were characterized morphologically and counted by adhering them to glass slides using the Cytospin system (Shandon, Southern Sewickley, PA) and then staining them by Kwik-Diff (Fisher Scientific, Boston MA). The adhered stained cells were air-dried and counted in a blinded manner under a light microscope.

### Murine HCL model of ALI

Mice were anesthetized with ketamine/xylazine (ketamine (125 mg/kg; Sanofi Winthrop Pharmaceuticals, New York, NY), xylazine (12.5 mg/kg; Phoenix Scientific, St. Joseph, MO) and 50 μl of hydrochloric acid (0.1 N HCl, pH 1.5, endotoxin free; Sigma-Aldrich) was instilled selectively into the left mainstem bronchus of anesthetized mice *via* a 24-gauge angiocatheter inserted intratracheally. 24 hours after acid instillation, bronchoalveolar lavage (BAL) was performed as described above and the BALF analyzed for total cell count and leukocyte differential as described above.

### FACS and cytospin analysis of neutrophils derived from bronchoalveolar lavage fluid following HCL or bleomycin treatment

Three days after bleomycin and 24h after HCL and LPS exposure, mice were anesthetized with an intraperitoneal injection of ketamine (125 mg/kg; Sanofi Winthrop Pharmaceuticals, New York, NY) and xylazine (12.5 mg/kg; Phoenix Scientific, St. Joseph, MO). The trachea was cannulated using a (24 gauge angiocatheter (Becton Dickinson), and BALF collected as described above. Filtered BALF and red blood cell–lysed blood cells were resuspended in FACS buffer (Mouse PBS, 2% fetal calf serum, 2 mM EDTA). Cells were stained with the two monoclonal specific monoclonal antibodies against neutrophils Anti-CD11B-PE and Ly6G-FITC (BD Bioscience, Woburn, MA) (20 min, 4 °C on ice). Cells were then washed and resuspended in 50 μl FACS buffer. All studies were performed on a FACS Calibur (Becton Dickinson, San Jose, CA), and data were analyzed with FlowJo software (Tree Star, Ashland, OR). To confirm the presence of neutrophils within the different flow cytometry samples, we separately characterized a sample from each group by adhering the cells to glass slides using the Cytospin system (Shandon, Southern Sewickley, PA) and then staining them by Kwik-Diff (Fisher Scientific, Boston MA). The adhered stained cells were air-dried and counted in a blinded manner under a light microscope.

### Cytokine analysis

#### Murine BALF

The Broncho-Alveolar Lavage fluid (BALF) supernatant from mice was collected following centrifugation (4 min at 7000g) and stored at -80°C. Inflammatory cytokine levels (IL1-β, IL-6 and TNF-α) in the BALF were determined as per manufacturer instructions using Enzyme Linked Immunosorbent Assay (ELISA) kits obtained from Biolegend, Inc, (San Diego, CA).

#### Rat plasma

Rats were anesthetized and bled into pre-chilled EDTA-microtainer tubes (Becton­Dickinson). The blood samples were processed to plasma which was stored in labeled 1.5-ml Eppendorf tubes at -80°C. Plasma samples were thawed to room temperature and assayed by ELISA for IL-6, IL-β and TNF-α (R&D Systems).

### Neutrophil migration assay

Neutrophil migration assays were performed using a modification of the Boyden chamber technique (20) using 24-well Transwell permeable supports (Corning, NY) with migration inserts (3.5μm pore size, 6.5 mm diameter). The bottom parts of the 24-well plate were pre-incubated for 60 minutes in serum-free DMEM containing 25 mM HEPES, pH 7.5 at 37°C (5% CO_2_, humidified) with either fVEGF (50nM, Genscript (Piscataway, NJ)), elastase (100 μg/ml, Fitzgerald, UK) + VEGF (10 ng/ml, Peprotech, Israel), or elastase 1 ug/ml μg/ml + VEGF (10 νg/ml) + PR1P (100 ug/ml). At the end of the 60-minute (digestion period) human neutrophils (IQ Biosciences, Alameda, CA) were plated onto the upper Transwell inserts at a density of 100,000 cells/insert and allowed to migrate towards the bottom chamber for two hours (migration period). Upon conclusion of the migration period cells that had migrated through the membrane were scraped and collected and washed once in PBS without Ca^2+^ and Mg^2+^, fixed with 100% pre-chilled methanol for 10 min, adhered to glass coverslips and then stained by H&E. Images of migrated cells were captured by light microscopy from six random regions of interest (10X magnification) and counted.

## Results

### PR1P mediates neutrophil migration *in vitro* by preventing VEGF degradation

**Figure 1:**
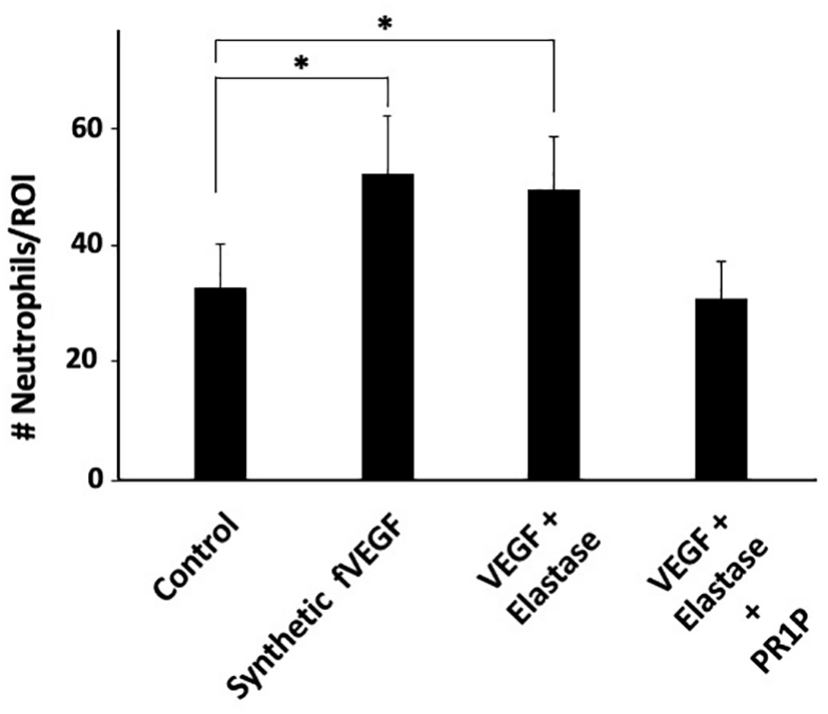
PR1P attenuates neutrophil migration *in vitro* by preventing VEGF degradation. Bar graph showing the quantification of neutrophils (# Neutrophils/Region of Interest (ROI)) during Transwell Plate Migration assay in which neutrophil migration (2h) was in response to indicated stimulants. ‘fVEGF’ was commercially synthesized based on a published amino acid sequence (20). ‘VEGF + Elastase’ and ‘VEGF + Elastase + PR1P’ were products of 30 min incubation of VEGF and elastase in presence or absence of PR1P, respectively. (n=6 repeated 3 times). Data are mean +/- SEM. *p<0.004.

fVEGF was recently shown to be chemoattractant to macrophages (21) and so we hypothesized that it similarly mediates neutrophil migration. Furthermore, we recently showed that PR1P protects VEGF from neutrophil-derived protease degradation by binding to the heparin binding domain (HBD) (27) on VEGF and preventing the formation of fVEGF. We therefore surmised that by binding to and stabilizing VEGF from proteolytic degradation into fVEGF, PR1P indirectly mediates neutrophil chemoattraction. To test this hypothesis, we modified an inflammatory cell migration assay (20) to test the effect of fVEGF and PR1P on neutrophil migration. As shown in **Figure 1**, both commercially synthesized fVEGF, as well as the product of VEGF pre-incubated with neutrophil derived elastase, induced migration of human neutrophils. In contrast, when PR1P was present during elastase treatment of VEGF, the ensuing product failed to induce neutrophil migration, indicating that PR1P prevents the generation of fVEGF and subsequent induction of neutrophil migration. Thus, PR1P indirectly influences neutrophil migration through its ability to protect VEGF from proteolytic degradation.

### PR1P limits neutrophil migration to sites of tissue injury in lung and gastro-intestinal injury models *in vivo*

**Figure 2:**
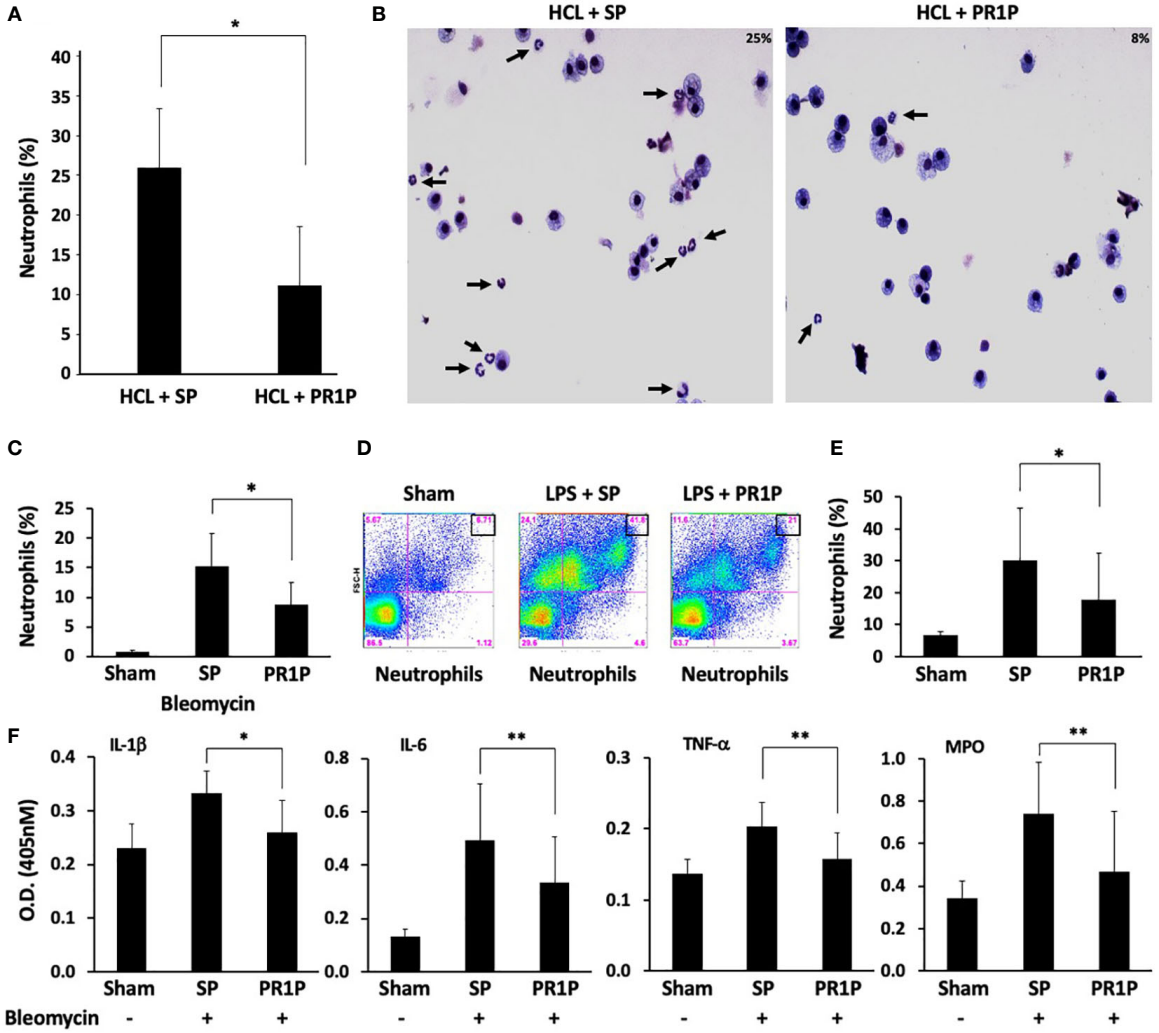
PR1P inhalation reduces neutrophil migration into lungs and lung inflammation following bleomycin, LPS and HCL injury in mice. **(A)** Bar graph showing percentage of neutrophils present (% of total cells in region of interest (ROI)) in BALF following IT administration of acid (HCl) and daily inhalation of PR1P or scrambled peptide (SP). n=8 repeated 3 times. *p<0.023 **(B)** Representative Diff-Quik stained cells from murine BALF described in **(A)** showing reduced neutrophils (black arrows) following PR1P treatment (25%) compared to SP (9%). **(C)** Bar graph showing percentage of neutrophils present (% of total cells in ROI) in BALF following IT administration of Bleomycin and daily inhalation of PR1P or scrambled peptide (SP). n=7 repeated 3 times *p<0.02. **(D)** Representative FACS analysis of cells from whole lung digests from mice treated with PR1P or SP after LPS injury showing reduced presence 24h after injury of neutrophils following PR1P inhalation. **(E)** Bar graph showing quantification of neutrophils (% of total cells) in whole lung cell digests from FACS experiments described in **(D)** n=8, repeated 3 times *p<0.02. **(F)** Bar graph showing quantification (Optical Density (OD) at 405 nm) of indicated cytokines or myeloperoxidase (MPO) activity in BALF following bleomycin injury in experiments described in **(A)** All data are mean + SEM. n=5. *p<0.004, **p<0.05.

Circulating neutrophils are often the first immune cells to arrive at sites of tissue injury and are thought to initiate the necessary inflammatory response required to restore tissue integrity (36). We therefore next sought to determine whether PR1P could be used to reduce neutrophil migration into injured lungs and/or GI tract following tissue injury in established animal models. Lung injury models were chosen to characterize the effect of PR1P on inflammation from diverse insults and injury mechanisms, and over different time courses. Specifically, lung injury was induced either by inhalation of lipopolysaccharide (LPS, a component of the bacterial cell wall and used as a potent trigger of acute lung inflammation whereby neutrophils migrate into the lungs within 4-24 hours (37)) or by intra-tracheal administration of bleomycin (38) (which induces acute inflammation and later fibrosis through a multi-factorial mechanism), or HCL (39) (also multifactorial in nature through damage to the alveolar-capillary membrane (40) resulting in immediate polymorphonuclear neutrophil (PMN) recruitment (41), pulmonary edema, and hypoxia (42)). In each of the lung injury models, tissue injury was followed by daily inhalation of PR1P or scrambled peptide (SP) until completion of each of the experiments according to the injury model (i.e. LPS and HCL for 24 hours, and bleomycin for 72 hours). FACS analyses of BALF following HCL- and bleomycin-induced lung injury revealed that inhaled PR1P significantly reduced toxin-induced neutrophil migration into the airways (i.e. BALF, HCl, **Figure 2A**, Bleomycin, **Figure 2C**). Representative Diff-Quik^©^ stained cells used in the FACS analyses following acid injury are shown in **Figure 2B**. Similarly, FACS analyses of cells from whole lung digests from LPS injured lungs show that inhaled PR1P significantly reduced the presence of migrated neutrophils into the lungs 24h after exposure to LPS (**Figures 2D-E**).

To determine whether reduced neutrophil migration into lungs correlates with reduced inflammation, we measured the levels of pro-inflammatory cytokines in BALF following bleomycin injury and found that inhaled PR1P significantly reduced levels of TNF-α, IL-1β, and, IL-6 compared to scrambled peptide (see **Figure 2F**). Furthermore, inhaled PR1P reduced myeloperoxidase (MPO) activity in lung tissue following bleomycin injury (**Figure 2F**). Together these data suggest that inhaled PR1P effectively reduces neutrophil migration into the lungs, and in so doing PR1P attenuates lung inflammation as determined by reduced cytokine levels and MPO activity.

### PR1P improves disease parameters in TNBS-induced colitis in

**Figure 3:**
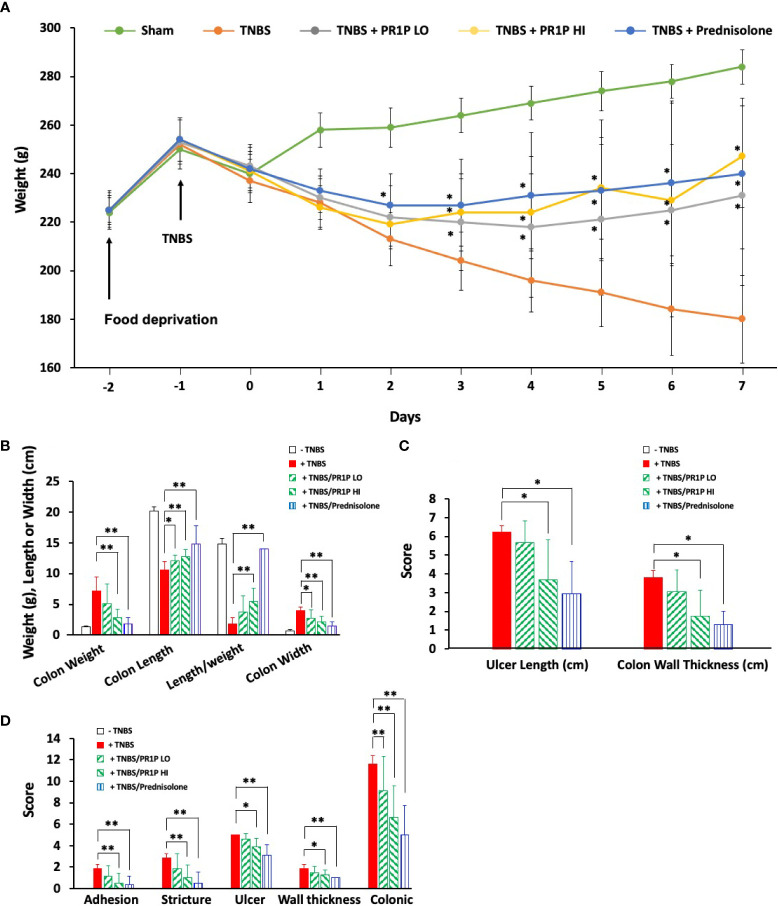
PR1P improves disease outcome parameters in TNBS-induced colitis in rats. **(A)** Line graph showing average daily weights of rats treated with daily low-dose PR1P (0.5 mg/kg IP, TNBS + PR1P LO) or high-dose PR1P (5 mg/kg IP, TNBS + PR1P HI) or prednisolone (10 mg/kg po, TNBS + Prednisolone) following induction of colitis from administration of intra-rectal TNBS. Sham animals received intra-rectal 50% ethanol (vehicle) to induce colitis and no treatments. TNBS group (+TNBS) received daily treatment with vehicles IP or po. *p<0.001 vs +TNBS group. **(B-D)** Bar graphs showing average colon weight, colon length, length/weight ratio and colon wall thickness **(B)**, *p<0.03, **p<0.004), ulcer length and colon wall thickness (**C**, *p<0.005) and average scores for adhesions, strictures, ulcer, wall thickness and colonic score using scoring system described in methods (**D**, *p<0.05, **p<0.009) from indicated treatment groups at the end of experiments described in **(A)**. Data in **(A-D)** are mean ± SEM. n=8 animals per group.

We next characterized the anti-inflammatory and potential therapeutic activity of PR1P in a TNBS-induced ulcerative colitis model (33). In this model, animals treated with TNBS develop acute colitis with bloody, mucus laden diarrhea and sustained weight loss that lasts UP TO three weeks (33). Induction of TNBS colitis (on Day 0) resulted in a decrease in body weight in all experimental groups (**Figure 3A**). Treatment with PR1P (low and high doses) and with prednisolone (which served as a positive control) significantly improved weights as early as Day 3 (PR1P, both doses) and Day 2 (Prednisolone) following injury as indicated. Upon study completion on Day 7, the colons of each animal were removed and measured for length and weight and examined macroscopically for ulcers. Both PR1P (high dose) and Prednisolone significantly attenuated TNBS-induced gain in colon weight (**Figure 3B**) and both low and high dose PR1P, and Prednisolone attenuated TNBS-induced colon shortening and widening (**Figure 3B**). Also, high dose PR1P and prednisolone each significantly reduced the TNBS-induced drop in the Length/Weight index (**Figure 3B**). Macroscopic examination of the colons also revealed that high-dose PR1P and prednisolone significantly attenuated increases in TNBS-induced ulcer length and colon wall thickness (**Figure 3C**) (Note the grades of Ulcer Lengths and Colon Wall Thickness in the control groups (i.e.-TNBS) are not visible in the graphs because their values are 0). Further macroscopic analysis and grading of the colons revealed that both high-dose PR1P and prednisolone significantly reduced TNBS-induced effects on adhesions, strictures, ulcers, and colon wall thickness (**Figure 3D**, note that each condition is graded 0-2, see Methods). Importantly, both low- and high-dose PR1P, and prednisolone significantly reduced the overall Colonic Score (Sums of the above conditions, Maximum score of 12, see Methods). These results suggest that PR1P significantly reduces critical disease indices following exposure to TNBS in rats.

### PR1P reduces inflammation in TNBS-induced rat colitis

**Figure 4:**
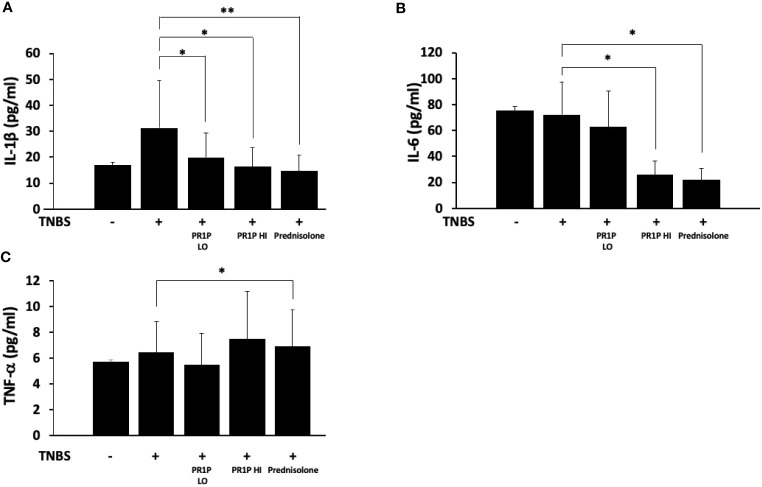
PR1P mediates inflammation in TNBS-induced colitis in rats. **(A-C)** Bar graphs showing quantification of IL-1β (**A,** *p<0.03, **p<0.003), IL-6 (**B,** *p<0.0001) and TNF-α (**C,** *p<0.04) in plasma on day 7 in rats treated with daily low-dose PR1P (0.5 mg/kg IP, PR1P-LO) or high-dose PR1P (5 mg/kg IP, PR1P-HI) or prednisolone (10 mg/kg) following induction of colitis from administration of intra-rectal TNBS. Control rats were treated with vehicle only. Data are mean ± SEM. n=8 animals per group.

To characterize the anti-inflammatory therapeutic potential of PR1P in TNBS-induced colitis, we quantified plasma levels of the pro-inflammatory mediators IL-1β, IL-6 and TNF-α on Day 7 following necropsy. As shown in **Figures 4A-C**, PR1P and prednisolone significantly reduced the TNBS-induced increases in plasma IL-1β and IL-6. Interestingly, high-dose PR1P treatment of TNBS-exposed animals led to a significant increase in plasma TNF-α (see Discussion). Together these results suggest that PR1P mediates the serum levels of critical pro-inflammatory cytokines during disease progression in TNBS-induced colitis in rats.

**Figure 5:**
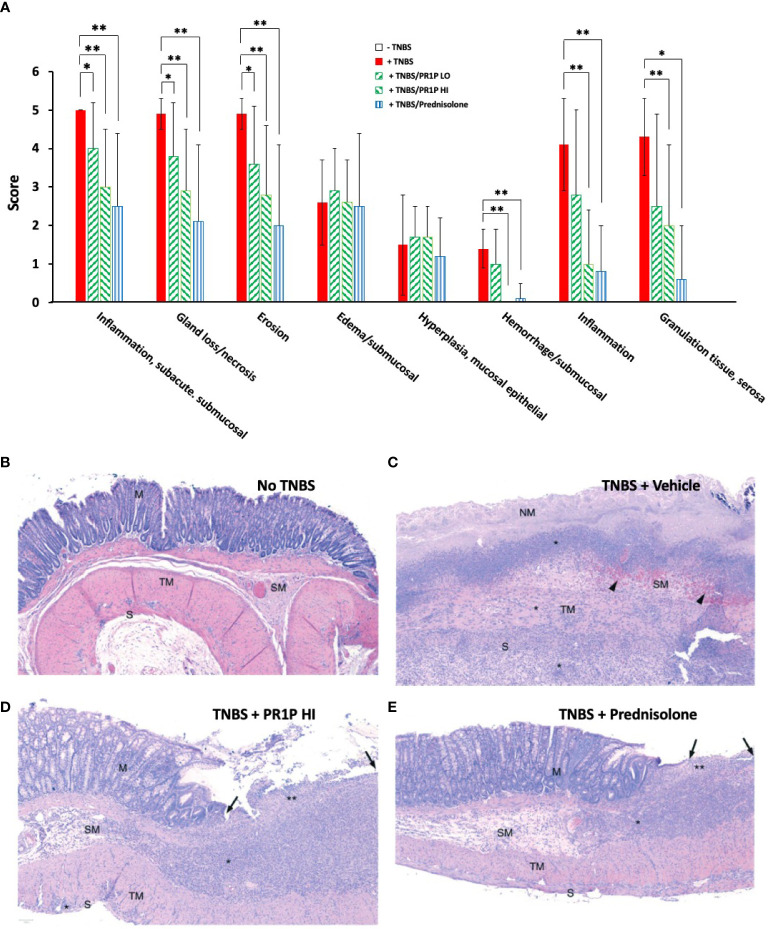
PR1P reduces disease and inflammation on histopathology in TNBS-induced colitis in rats. **(A)** Bar graphs showing Average Scores (Score) of indicated lesions from histology sections of the colon of rats 7 days after intra-rectal administration of TNBS (or carrier) and daily treatment of PR1P (0.5 or 5 mg/kg IP) or Prednisolone (10 mg/kg po) as indicated (See Methods), Data are mean + SEM. *p<0.04, **p<0.005. (n=8). **(B-E)** Representative images of H&E stained colon sections (100X) obtained from rats on day 7 following experiments described in **(A)**. **(B)** Representative image from Naïve animal. No histologic lesions observed in captured region. The mucosal glands (M) are within normal limits, as are the submucosa (SM), tunica muscularis externa (TM), and the serosa (S). **(C)** Representative image from animal with TNBS-induced colitis treated with vehicle (TNBS + Vehicle). No normal mucosa remains in captured region; all mucosa exhibits coagulative necrosis (NM: necrotic mucosa), with complete loss of the surface epithelium, consistent with erosion. A thick band of inflammatory cells (*) are visible at the mucosal-submucosal (SM) border. The submucosa is edema-expanded and contains abundant hemorrhage and erythrophagocytic macrophages (black arrowheads). Inflammatory cells (*) also extend transmurally through the tunica musuclaris (TM) and serosa (S) into the serosal granulation tissue visible at the bottom of the image. **(D)** Representative image from animal with TNBS-induced colitis treated with high dose PR1P (5 mg/kg, TNBS + PR1P HI). The right side of the image exhibits inflammatory cell infiltration and gland loss (**) with surface epithelial loss (erosion; black arrows). The adjacent mucosa (M) is hyperplastic, with elongated colonic glands observed. The submucosa (SM) is expanded by edema and inflammatory cell infiltration (*). Very mild extension of inflammation (*) is seen through the tunica muscularis (TM) and into the serosa (S). **(E)** Representative image from animal with TNBS-induced colitis treated with Prednisolone (TNBS + Prednisolone). Inflammatory cell infiltration with gland loss (**) and erosion (black arrows) are mild; the adjacent mucosa (M) exhibits reactive hyperplasia. There is expansion of the submucosa (SM) by edema (clear/pale pink area) and inflammatory cell infiltration (*). Inflammatory cells are generally limited to the mucosal and submucosa with minimal involvement of the tunica muscularis externa (TM) and serosa (S).

Histopathologic findings in this study were consistent with the TNBS-induced rat colitis model (33), and administration of TNBS was associated with the highest mean histopathology scores for most features, including sub/mucosal inflammation, necrosis, erosion, hemorrhage, transmural inflammation, and serosal granulation tissue (**Figure 5A**). As shown in **Figure 5A**, both the low- and high dose PR1P, and prednisolone, significantly reduced the effects of TNBS in our model on many features scored including inflammation, gland loss/necrosis, erosion/ulceration, sub-mucosal hemorrhage, and granulation tissue. Representative images of H&E stained colon sections (Day 7) from naïve animals and those exposed to TNBS and indicated treatment protocols are shown in **Figures 5B-E** and illustrate the effect of PR1P on disease development. It is known that neutrophil migration into the colon begins immediately after injury, peaks at 24 hours, and drops to approximately 50% and 20% of maximum by days 3 and 10, respectively (43). Representative H&E stained images of colonic tissue sections from animals on Day 7 are shown in **Figures 5B-E** and illustrate the changes reported in **Figure 5A** showing PR1P dose-associated reductions in histopathologic features that are similar to prednisolone treated animals.

## Discussion

Here we show that VEGF degradation products generated *via* VEGF proteolysis by neutrophil elastase are chemoattractant to neutrophils. We subsequently characterized the ability of a novel VEGF binding and stabilizing peptide we developed to mediate endogenous VEGF signaling and inflammation in three murine models of acute lung injury and in a rat ulcerative colitis model in which tissue injuries were induced by diverse mechanisms. Importantly, by binding VEGF within the VEGF heparin-binding domain where proteases are known to bind VEGF (44, 45), PR1P prevents VEGF degradation (46). As we hypothesized, PR1P reduced neutrophil migration into injured tissue and reduced local inflammation following tissue injury. Our results underscore the importance of VEGF in mediating inflammation following tissue injury, and support its importance in maintaining and restoring tissue to health. Furthermore, these studies support a potential role for PR1P in treating diseases commonly caused by tissue injury and characterized by inflammation and VEGF dysregulation.

We chose ARDS and UC animal models to characterize the ability of PR1P to improve outcome because both these diseases are induced by tissue injury and are characterized by local inflammation and VEGF dysregulation (2, 15, 19). Despite widely disparate injury mechanisms, all four models we tested (LPS, acid, bleomycin and TNBS) are known to result in early neutrophil migration into injured tissue and acute inflammation (47–49). Neutrophils are central to innate immunity (50), and are implicated in the onset and progression of many inflammatory diseases, including ARDS (24) and UC (9, 51). Neutrophils are often the first immune cells to arrive at sites of tissue injury and mediate both the initiation and resolution of the inflammatory response that is crucial in returning injured tissue to health (36). Production and release of pro-inflammatory cytokines including tumor necrosis factor (TNF)-α, interleukin (IL)-1β and interleukin (IL)-6 (52), secreted by a variety of immune cells including neutrophils, macrophages, T- and B- cells, dendritic cells, and vascular endothelial cells (53) also follow tissue injury, and together with the appearance of neutrophils in both ARDS and IBD, are central to the initiation and maintenance of inflammation (53). VEGF dysregulation following tissue injury is due to the release by inflammatory cells, including neutrophils, of serine proteases including elastase and plasmin (54, 55) which degrade VEGF into smaller VEGF isomers (VEGF degradation products, fVEGF) with altered VEGF receptor binding and signaling properties (20, 21). Thus, it is not surprising that injury to most tissues, irrespective of mechanism, results in acute inflammation and VEGF dysregulation.

Although it is well documented that VEGF levels and VEGF signaling are dynamic during both ARDS and IBD: it is not clear how this important growth and angiogenic factor mediates disease outcome during illness (15, 18, 19). ARDS is an acute inflammatory disease resulting from diverse direct and indirect insults to the lungs (1) and is characterized by fulminant acute respiratory failure requiring mechanical ventilation and prolonged ICU admission (3). There are no medicines to cure ARDS, and mortality remains high (56). VEGF is widely thought to play a protective role during ARDS progression as VEGF has been shown to mediate the survival of lung airway epithelial cells, and the repair of damaged alveolar capillary membrane barriers thereby reducing pulmonary edema (17, 18). Decreased VEGF levels in the lungs as measured in broncho-alveolar lavage fluid and in exhaled breath condensate (57) of critically ill patients with ARDS are associated with poor prognosis (58, 59). The decline in VEGF levels in the lungs in patients with ARDS is thought to be due to degradation of VEGF by inflammatory cell derived proteases in the alveoli (20, 21). Interestingly, serum concentrations of VEGF increase early in ARDS and return to normal if patients recover (60, 61). This dynamic is thought to be due to release of VEGF from the lungs into the systemic circulation due to impaired epithelial and endothelial barriers (61).

IBD occurs in two forms, ulcerative colitis (UC) and Crohn’s disease, which are generally, but not always, distinguished by the portion of the gastrointestinal tract affected by disease (62). IBD is thought to be a consequence of inappropriate and unrelenting activation of the mucosal immune system (63) whereby inflammatory cells including neutrophils, secrete proteases which impair mucosal repair through inhibition of epithelial cell proliferation and through degradation of adhesion and signaling molecules including adherens junction protein, E-cadherin, elastin, collagen, and MMP inhibitors that participate in the maintenance of the intestinal barrier (25, 64–68). In a similar fashion to ARDS, systemic levels of VEGF may elevate during disease due to the release of VEGF from the GI tract from vascular endothelial breakdown (69). Chronic inflammation in IBD is due in part to ongoing production of cytokines by inflammatory cells. There are several drugs available to treat IBD that each target the inflammatory process *via* different mechanisms and include amino-salicylates, corticosteroids, immunomodulators, and anti-TNF-α antibodies. Despite these multiple medical options, proctocolectomy to remove irreversibly diseased intestine is performed in 30% of patients with IBD (9, 70, 71). Thus, the lack of a medical therapy to treat ARDS, and the fact that one-third of patients with IBD do not respond to therapy (71) highlight the need for the development of an alternative therapeutic such as PR1P to treat these common and debilitating diseases that are characterized by inflammation and VEGF dysregulation.

The mechanism of action of PR1P is through its binding to VEGF within the VEGF Heparin Binding Domain (HBD) thereby impeding the binding of VEGF by proteolytic enzymes. In so doing, PR1P prevents degradation of VEGF into fVEGF, which we demonstrate is a neutrophil chemoattractant, and which therefore stimulates further inflammation. It is intriguing that PR1P had opposite effects on TNF-α levels in the lung and GI tract, respectively, at the different timepoints sampled in our models. Cytokines play an important role in dysregulated immune responses and an imbalance between pro- and anti-inflammatory cytokines can have deleterious effects on disease outcome. Patterns of pro- and anti-inflammatory cytokine levels are dynamic over the course of specific diseases and cytokine levels reflect animal- and organ- specific stages of illness. Also, TNF- α was recently shown to have anti-inflammatory properties in a dextran sodium sulfate (DSS)–induced colitis model (72). Regardless of mechanism, results of these studies support our own and point to a critical role for PR1P in mediating inflammation. VEGF therapy involves strategies to deliver VEGF (through direct delivery of VEGF or modified VEGF), or *via* gene therapy to increase local tissue VEGF concentrations (73). Although successful in animal models, VEGF therapy has proven unsuccessful in human studies for many reasons, including that the treatment of acute or chronically inflamed tissue with VEGF likely leads to rapid VEGF degradation and increased local concentrations of fVEGF due to elevated concentration of proteases. This therapeutic approach fails to upregulate VEGF signaling locally and instead likely stimulates further inflammation. Thus, PR1P is fundamentally different than conventional VEGF therapy as PR1P stabilizes endogenous VEGF within local tissue microenvironments, preserves endogenous VEGF signaling, and reduces the production of fVEGF.

As noted, PR1P binds VEGF within the VEGF HBD (46) thereby competing with proteases for binding to VEGF. The HBD is an important carboxy-terminal region on VEGF that plays a critical role in VEGF signaling and therefore may serve as an important target for drug development. Disparities in HBD expression between alternate splice forms of different VEGF isoforms result in subtle variations in downstream signaling (74, 75). For example, proteolytic degradation of the whole or parts of the carboxy-terminal region of the VEGF HBD significantly reduced VEGF-induced mitosis in human vascular endothelial cells (22, 76). Furthermore, Kurtagic et al. showed that neutrophil-derived elastase (NE) cleaved VEGF within internal regions of the N-terminus of VEGF in addition to regions at the C-terminus (20, 21). They subsequently showed that NE generated VEGF fragments (fVEGF) demonstrated higher binding affinity to VEGF Receptor 1 (VEGFR1) compared to VEGFR2 (20, 21) which is significant because VEGFR1 is expressed on neutrophils (77). Thus, in addition to attracting neutrophils into sites of tissue injury, fVEGF also likely upregulates neutrophil activity once present within the inflamed tissue. Collectively, these findings provide key evidence that the VEGF HBD may play a central role in mediating VEGF signaling during both health and in disease. Keyt et al. showed that chronic wounds are associated with a high concentration of proteases, elevated levels of fVEGF, and decreased VEGF activity (44). Treatment of chronic wounds with a mutant VEGF containing a modified HBD that prevents plasmin binding to VEGF resulted in increased VEGF stability within the wound that resulted in increased angiogenesis (in surrounding wound tissue) and improved wound healing (44). It is well known that VEGF signaling controls a wide variety of biological and functional outcomes (78, 79) that are important in maintenance of tissue health and healing (12, 80). Although the signaling and binding properties of fVEGF are not fully elucidated, our data and those of others suggest that fVEGF may serve as a point of convergence in VEGF signaling that may determine whether tissues begin to heal and remodel or remain inflamed.

In summary, we investigated the potential link between neutrophils, often the first immune cell to arrive at the site of tissue injury from multiple disparate direct and indirect insults, and VEGF, a ubiquitous signaling molecule implicated in mediating healing of injured tissues. We discovered that fVEGF, a product of VEGF degradation by inflammatory cell derived proteases, is chemoattractant to neutrophils. Furthermore, we showed that PR1P, a VEGF binding and stabilizing peptide reduced neutrophil migration into injured tissue and inflammation in animal models of ARDS and UC. PR1P is a distinct alternative to conventional VEGF therapy. Our studies underscore the importance of VEGF in mediating tissue during both health and disease and also point to fVEGF serving as a point of convergence in VEGF signaling that may determine whether tissues begin to heal and remodel or remain inflamed. To date there has been little work done in developing pharmaceuticals for ARDS and UC that target VEGF. Our work suggests that drug development to support PR1P or other molecules targeting fVEGF biology may prove impactful in treating diseases like ARDS or UC that are characterized by VEGF dysregulation and inflammation.

## Data availability statement

The raw data supporting the conclusions of this article will be made available by the authors, without undue reservation.

## Ethics statement

The animal study was reviewed and approved by Boston Children’s Hospital (BCH) Institutional Animal Care and Use Committee (IACUC) Protocol Title: PR1P therapy for murine emphysema 20-05-4174R.

## Author contributions

AA and BM designed and performed the experiments, analyzed the data, and wrote the manuscript. VK MP, CK and SL assisted in performing lung’s experiments. JB assisted in performing UC experiments. All authors contributed to the article and approved the submitted version.
